# Medication review versus usual care to improve drug therapies in older inpatients not admitted to geriatric wards: a quasi-experimental study (RASP-IGCT)

**DOI:** 10.1186/s12877-018-0843-y

**Published:** 2018-07-03

**Authors:** Lorenz Van der Linden, Julie Hias, Lisa Dreessen, Koen Milisen, Johan Flamaing, Isabel Spriet, Jos Tournoy

**Affiliations:** 1 Pharmacy Department, University Hospitals Leuven, Herestraat 49, 3000 Louvain, Belgium; 2 Department of Pharmaceutical and Pharmacological Sciences, KU Leuven, Louvain, Belgium; 3 Department of Geriatric Medicine, University Hospital of Brussels, Brussels, Belgium; 4 Department of Public Health and Primary Care, Health Services and Nursing Research, KU Leuven, Louvain, Belgium; 5 Department of Geriatric Medicine, University Hospitals Leuven, Louvain, Belgium; 6 Department of Chronic Diseases, Metabolism and Ageing, KU Leuven, Louvain, Belgium

**Keywords:** IGCT, Older inpatients, Medication review, PIM, Screening tool, Drug use, Polypharmacy

## Abstract

**Background:**

Interdisciplinary geriatric consultation teams (IGCT) are regularly requested to provide comprehensive geriatric assessments in older inpatients. Our primary aim was to evaluate whether medication reviews increased the number of IGCT-provided drug-related recommendations. Secondary aims were to reduce the number of potentially inappropriate medications (PIMs), and to identify the acceptance rate of and determinants for the number of recommendations.

**Methods:**

A before-after study was performed in older inpatients not admitted to acute geriatric wards. The before cohort received usual care (UC); the after cohort was subjected to the intervention (I), consisting of a systematic medication review, based on but not limited to the RASP (Rationalization of Home Medication by an Adjusted STOPP in Older Patients) list. The primary outcome measure was the number of IGCT-provided drug-related recommendations. Age, sex, Charlson Comorbidity Index, creatinine clearance and serum creatinine were ascertained upon enrolment. Following variables were determined on admission and at discharge: number of drugs and number as well as type of RASP-identified PIMs. Acceptance by ward-based physicians was also determined. Poisson regression was performed to identify determinants for the primary outcome measure.

**Results:**

Fifty-nine participants were enrolled (n_UC_ = 29; n_I_ = 30). The intervention increased the number of drug-related recommendations from a median of 0 (IQR: 0–1) to 8 (IQR: 6.75–10) (*p* < 0.001). The median number of accepted recommendations differed significantly as well (UC vs. I: 0.0 (0.0–0.5) vs. 3.0 (0.0–5.3); *p* < 0.001). In the intervention cohort, patients were discharged with fewer drugs compared to admission (UC vs. I: 108.5%, IQR: 100.0–135.8% vs. 92%, IQR: 80.5–103.5%; *p* = 0.002). More RASP PIMs were discontinued in the intervention cohort, with a mean difference of 1.49 RASP PIMs (95% confidence interval (CI): 0.70, 2.23; *p* < 0.001). Regression analysis identified two determinants: allocation to the intervention cohort with an incidence rate ratio (IRR) of 14.1 (95% CI: 8.30, 23.8) and the number of preadmission drugs with an IRR of 1.06 (95% CI: 1.03, 1.09).

**Conclusions:**

A structured medication review as part of usual IGCT care may contribute to an increased detection of drug-related problems and help to further reduce polypharmacy in older inpatients, not admitted to acute geriatric care wards.

**Trial registration:**

NCT02165618, retrospectively registered June 17, 2014.

## Background

Inappropriate drug use remains common in older persons and has been associated with a lower quality of life, increased health-care utilization and an increased readmission risk [1]. Causes are many and frequently complex, yet have been shown to be at least partially amenable to change [1–8].

Multiple comprehensive interventions have already been demonstrated to improve the quality of prescribing and to reduce the number of potentially inappropriate medications (PIMs). Most interventions have been limited to the inpatient setting, which might partially be explained by the provision of increased monitoring during hospital stay and also by higher baseline risk of the hospitalized patient population for adverse (drug) events, resulting in a relatively lower needed sample size for interventional studies [9]. In several trials, such interventions relied on the addition of ward-based hospital pharmacists to the multidisciplinary care team [4]. Hospital pharmacists provided several services, such as medication reconciliation on admission, supporting the implementation and adjustment of clinical decision support systems (CDSS), patient education and medication review [2, 5, 8, 10]. Pharmacist-led interventions affect clinical outcome; a decrease in the number of unplanned drug-related readmissions and emergency department visits has been observed, as concluded in recent meta-analyses [1, 4]. More data are still needed however to corroborate previous findings on the perceived benefit of medication review in older adults and also to further confirm the perceived benefits in specific subgroups [1, 4, 11, 12].

To ascertain the appropriateness of drug therapies in older adults, several screening tools have been made available [13]. We have developed the RASP (Rationalization of Home Medication by an Adjusted STOPP in Older Patients) list, which was subsequently investigated as part of a pharmacist-led medication review in a controlled trial which took place on several acute geriatric wards in a large teaching hospital [2, 13]. This approach was shown to improve the quality of prescribing without increasing harm in a very old, predominantly octogenarian, inpatient population [2]. The intervention was however restricted to patients admitted to dedicated acute geriatric care units, limiting the benefits to a select subgroup of older adults. In many hospitals the majority of octogenarians are admitted to non-geriatric wards rather than to acute geriatric care units.

Previous reports have shown that a comprehensive geriatric assessment (CGA) by the interdisciplinary geriatric consultation team (IGCT) improved care in selected inpatient populations [14–16]. Considering the manner in which the CGA is offered, it is still not completely elucidated whether to opt for a fully decentralized assessment of geriatric patients, to develop a co-management model or to transfer and admit high risk older patients to dedicated acute geriatric wards [17–19]. It has furthermore not been established whether IGCT involvement might lead to an improvement in drug use in older patients. Dalleur et al. performed a randomized controlled trial to investigate this subject. The authors concluded that the use of the STOPP criteria by trained geriatricians, as members of the IGCT, led to a doubling of the reduction of PIMs in older inpatients admitted to non-geriatric medical units in a Belgian hospital [20]. The decentral IGCT team composition and provided care have however been shown to be highly heterogeneous and results by Dalleur et al. can hence not be readily extrapolated [20, 21].

As of 2014, Belgian law has provided a fixed budget for a minimum of two full time equivalents (mostly nursing staff), excluding fees for geriatricians, who are to be financed through other means. While improving care in older inpatients is central to the IGCT care model, the specific tasks and composition of IGCTs differ substantially across hospitals [21]. In our hospital, a medication review is not performed systematically by the IGCT. Frequently, the IGCT has rather recommended to perform additional blood tests, to screen for and treat low haematocrit or haemoglobin, to organize transfer to a rehabilitation centre or to organize professional home care [14].

The aim of this pilot study (RASP-IGCT) was to evaluate whether a medication review carried out by non-geriatricians using the RASP list and provided within the context of a CGA might result in an increase of drug-related recommendations in frail older patients, who had been evaluated by the IGCT and who were admitted to non-geriatric wards. Furthermore, this study aimed to evaluate the acceptance of the recommendations, to reduce the number of PIMs and to identify significant determinants for the number of IGCT provided drug-related recommendations.

## Methods

### Design and setting

The RASP-IGCT study was designed as a monocentric before-after study in older inpatients, who were not admitted to acute geriatric wards. This quasi-experimental design was used, due to the potential risk of contamination bias as the same IGCT would be employed in the before (usual care) and after (intervention) cohorts. Patients enrolled in January 2014 were consecutively admitted to the usual care cohort. In February 2014, patients were consecutively admitted to the intervention cohort, in which they were subjected to a systematic medication review. Ward-based physicians were blind to the study design.

The study took place in a 2000-bed teaching hospital, the University Hospitals of Leuven, Belgium. The study was approved by the local Ethics Committee. Patients were considered for inclusion if written informed consent was provided by the patients, or by their relatives in case of them being unable to provide consent (ClinicalTrials.gov identifier NCT02165618).

### Study participants

Initial assessment of the patient’s geriatric profile was performed by ward-based nurses as part of the patient assessment upon admission. The Flemish version of the six-item Triage Risk Screening Tool was used to ascertain the geriatric risk profile. A minimum score of 2 and active agreement of the treating non-geriatrician physician was needed to subsequently alert the IGCT [22].

Dutch-speaking patients, for whom an IGCT consult was requested, were eligible for inclusion if the following inclusion criteria were met: informed consent, age of 70 years or older, admission to a non-geriatric nursing ward and an enquiry for consultation from the geriatric consultation team. Patients were excluded from study participation if any of the following was applicable: no drug therapies upon admission to the hospital, presence of a terminal illness, transferal from another hospital or an acute geriatric ward, and an intensive care unit admission during hospital stay.

### Baseline characteristics

Patient characteristics and baseline variables were collected upon enrolment. Following data were gathered from the admission files: age, sex, weight, number of preadmission drugs, Charlson Comorbidity Index (CCI), serum creatinine concentration (mg/dl) and estimated creatinine clearance and glomerular filtration rate according to the Cockcroft-Gault and CKD-EPI equations, respectively [23].

Furthermore, the best possible preadmission medication list was collected for all patients in a standardized manner [2, 24]. When patients were not able to adequately respond to the questions, or when their cognitive state made the answers unreliable, their family, caretakers, community pharmacist or general practitioner were contacted to verify the correctness of the medication list. If the exact medication list remained unclear, the information obtained by the general practitioner was considered to be the correct one. The RASP list was subsequently applied to the preadmission medication list; potentially inappropriate medications (PIMs) identified by the RASP list were defined as RASP PIMs. Number and identity of RASP PIMs on admission were then determined.

### Usual care

Usual care was the care provided in the pre-implementation cohort by the IGCT. The IGCT performed a CGA and provided recommendations to the ward-based physician in order to improve geriatric care. A structured medication review was not applied systematically.

The team consisted of two geriatricians (JF and KF), three nurses, a social worker, two occupational therapists, and a physiotherapist. Direct patient contact was mainly carried out by the nurses. One geriatrician was present during the daily meetings, that took place on weekdays and during which all active cases were discussed. All recommendations were then added to the patient’s file.

### Intervention

The study was performed by a postgraduate pharmacist (JH) and an final year undergraduate physician (LD). Both investigators were trained in the use of the RASP list and were subsequently monitored by a senior clinical pharmacist (LVDL). The reproducibility, by which both JH and LD applied the RASP, was determined by calculating Cohen’s kappa on a set of 20 anonymized sample patient records (Cohen’s kappa: < 0.00: no agreement, 0.00–0.20: slight agreement, 0.21–0.40: fair agreement, 0.41–0.60: moderate, 0.61–0.80: favourable, 0.81–1.00: almost perfect agreement) [25]. We aimed to limit the time for medication review, which included patient screening and actual enrolment, to 30 min per patient.

The investigators performed a systematic medication review, taking into account both the preadmission medication list and the prescriptions that were present in the patient’s electronic prescription file at the moment of IGCT assessment. The medication review was performed once and within 24 h after the IGCT request. Investigators used the RASP list as basis for the medication review, but were not limited to this screening tool and were allowed on a case by case basis to deviate and base their recommendations on other sources or approaches such as the Garfinkel method or the tool described by Scott et al., with a strong emphasis on identifying an indication for each drug therapy [6, 7]. Investigators were instructed beforehand by a senior clinical pharmacist, who also provided clinical supervision thereafter.

Investigators reported the drug-related recommendations, both RASP-based and non-RASP-based, directly to the IGCT during the daily meetings. Drug-related recommendations were then adopted in the IGCT notes, which were shared in the electronic patient file with other health-care professionals.

### Outcome measures

The primary outcome measure was defined as the number of medication-related recommendations provided by the IGCT team. All IGCT recommendations were assessed one by one. Drug-related recommendations were subsequently identified and investigators verified whether they corresponded to any of the RASP criteria (i.e. RASP-based recommendations).

Following variables were collected as well: the identity and the number of RASP identified PIMs at discharge; the number of other medication-related (i.e. non-RASP based) recommendations; the number of drugs at discharge; the number of discontinued RASP PIMS at discharge. Furthermore, the ratio of the number of drugs at discharge to the number of drugs on admission was also calculated.

Acceptance of drug-related recommendations was defined as the agreement and subsequent change of prescription by the treating physician within 72 h after having been communicated to the physician by the IGCT. It was moreover documented whether IGCT recommendations had been adopted into the discharge letter.

### Statistical analysis

Normality of continuous variables was ascertained by performing the Kolmogorov-Smirnov test and by manual, visual inspection of the histograms. Parametric variables were characterized by mean (standard deviation, SD) and non-parametric data by median (interquartile range, IQR = Q1, Q3). Proportions and counts were represented as *n* (%). Categorical variables, parametric data and non-parametric data were compared using Fisher Exact test, Student’s *t* test and the Mann-Whitney *U* test respectively.

A Poisson loglinear regression analysis was performed to identify determinants for the primary outcome measure of IGCT provided drug-related measures. First, an unadjusted model was developed with the intervention vs. usual care as the sole variable (model A). Then, a backward stepwise approach was followed to reach a parsimonious model (model B), in which a main effect analysis was done. Selection of predictors was based on significance shown in an univariate analysis; following variables were tested: age, sex, CCI total score, CCI components, renal function (according to CG and to CKD-EPI), surgical vs internal medicine ward, number of preadmission drugs and the number of preadmission RASP PIMs. Additionally, a low Akaike information criterion (AIC) value was preferred.

A total sample size of 44 patients (i.e. 2 × 22) was estimated to detect a median difference of two recommendations with the Mann Whitney U test, with alpha and beta defined as 0.05 and 0.20 respectively. Due to the practical and explorative nature of the study, we aimed to enrol 30 patients per cohort (usual care and intervention).

All statistical tests were two-tailed and statistical significance was set at *p* < 0.05. Statistical analysis was performed using SPSS (Statistical Package for Social Sciences; IBM Corp. Released 2013. IBM SPSS Statistics for Windows, Version 22.0. Armonk, NY: IBM Corp).

## Results

Both investigators (JH and LD) showed a favourable reproducible approach in the training set of patient cases, respectively scoring a Cohen’s kappa of 0.72 and 0.73.

**Fig. 1:**
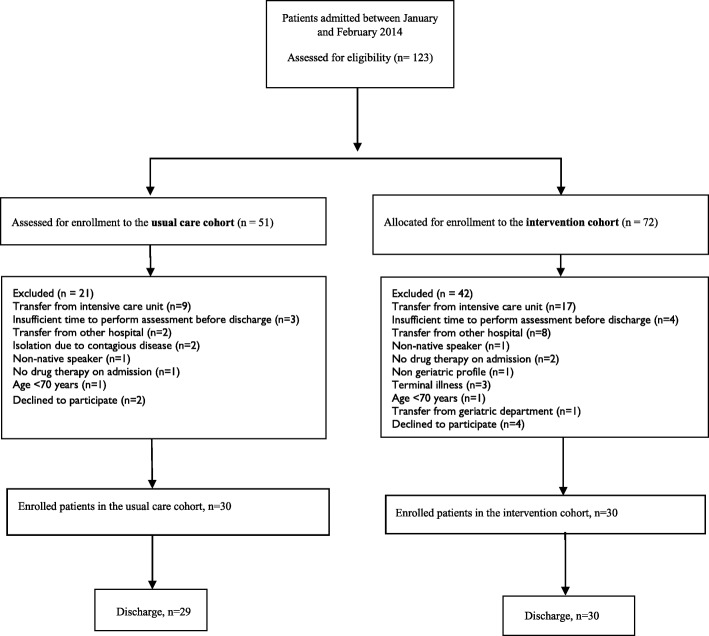
Flowchart of enrolled patients

In total, 60 patients were enrolled consecutively of whom 59 were included in the analysis of the primary outcome measure. One patient was excluded from analysis due to the institution of end-of-life care during hospital stay which precluded any IGCT counselling. Detailed information on patient flow through the study is depicted in Fig. 1.

Patient characteristics did not differ significantly between both cohorts. Average age-adjusted CCI was 7.12 (95% confidence interval (CI): 6.39, 7.84). Patients were drawn from 20 nursing wards, covering a total of 14 medical and surgical disciplines. More information has been provided in Table 1. Most common disciplines were the trauma ward (19/59) and cardiology units (15/59). In total, 24 and 35 patients were admitted to a surgical or medical unit, respectively. Twenty-three patients (23/59) were admitted to the hospital due to a fall as the main reason or as one of multiple reasons for admission. There was no difference seen in the number or type of RASP PIMs at baseline in the usual care vs. intervention patients, except a higher frequency of potentially inadequate inhalation in COPD in the intervention cohort (usual care vs. intervention: 0/29 vs 6/30, *p* = 0.024). Other prevalent pre-admission RASP PIMs have been summarized in Table 2.

**Table 1:** Baseline patient characteristics

	Usual Care (*n* = 29)	Intervention (*n* = 30)	*p*-value
Age (years) (median, IQR)	83 (79–86)	83 (78–88)	0.933
Weight (kg) (median, IQR)	72 (63–81)	71 (60–81)	0.814
Sex (male/female)	13/16	13/17	1.000
Number of preadmission drugs (median, IQR)	7.0 (6.0–11.0)	9.5 (5.8–13)	0.199
Patients with at least 1 RASP PIM (%)	100%	96.7%	1.000
Patients with > 5 drugs (%)	82.8%	90.0%	0.472
Patients with > 10 drugs (%)	37.9%	50%	0.435
Number of preadmission RASP PIMs (median, IQR)	3.0 (2.0–5.0)	3.5 (2.0–5.0)	0.437
Age-adjusted Charlson Comorbidity Index (median, IQR)	6 (5–8)	7 (5–8)	0.145
Serum creatinine (mg/dl) (median, IQR)	0.97 (0.78–1.41)	1.17 (0.90–1.49)	0.285
eGFR CKD-EPI (ml/min/1,73m^2^) (median, IQR)	63 (43–80)	50 (38–68)	0.148
CrCl CG (ml/min) (median, IQR)	52 (42–64)	43 (32–54)	0.159

*PIM* potentially inappropriate medications

*RASP* Rationalization of Home Medication by an Adjusted STOPP in Older Patients

*eGFR CKD-EPI* Estimated Glomerular Filtration Rate Chronic Kidney Disease Epidemiology Collaboration equation

*CrCl (CG)* Creatinine Clearance Estimated by the Cockcroft-Gault equation

**Table 2:** Prevalent pre-admission RASP PIMs, other than potentially inadequate inhalation in COPD

RASP item	Usual Care (*n* = 29)	Intervention (*n* = 30)	*p* value
Prolonged use of PPI or H2RA in peptic ulcer disease	11	8	0.412
Prolonged use of benzodiazepines, zolpidem, zopiclone or zaleplon	7	11	0.399
Prolonged use of antidepressants	5	10	0.233
Sedatives and hypnotics: benzodiazepines/zaleplon/zolpidem/zopiclone in fallers	9	6	0.382
Inadequate use of inhalation therapy in patients with moderate/severe cognitive and/or functional disability	6	8	0.761
Antidepressants in fallers	8	5	0.360
Vitamins, minerals and trace elements without documented deficiency	7	6	0.761
Additional dietary supplements without any documented need, not described in other RASP criteria (e.g. glucosamine)	2	6	0.254
Venotropic drugs	3	4	1.000
Antihypertensive drugs in the presence of postural hypotension	5	1	0.103

*COPD* chronic obstructive pulmonary disease

*RASP* Rationalization of Home Medication by an Adjusted STOPP in Older Patients

*PIM* potentially inappropriate medication

*PPI* proton pump inhibitor

*H2RA* histamine-2 receptor antagonist

In total, 254 drug-related recommendations were provided by the IGCT, of which 94.1% occurred in the intervention cohort. Due to the RASP-based intervention, the number of drug-related recommendations increased from a median of 0 (IQR: 0–1) to 8 (IQR: 6.75–10) per patient (*p* < 0.001).

The median number of accepted recommendations differed significantly in favor of the intervention cohort group (usual care vs. intervention: 0.0 (0.0–0.5) vs. 3.0 (0.0–5.3), *p* < 0.001). The median acceptance rate did not differ between both cohorts (usual care vs. intervention: 45% vs 50%, *p* = 0.140).

At least one IGCT recommendation was adopted into the discharge letter in more patients in the intervention (24/29) than in the usual care cohort (4/29) (*p* < 0.001). In the intervention cohort, 47.2% (IQR: 33.2–50.0%) of the drug-related recommendations was based on the RASP list, which was significantly more than in usual care patients (0.0%, IQR: 0.0–0.0%) (*p* < 0.001). Additionally, other drug-related recommendations, that were not based on RASP list, were observed more frequently in intervention patients (median (IQR): usual care vs. intervention: 0.0 (0.0–0.0) vs. 3.0 (2.0–5.0), *P* < 0.001).

In the intervention cohort, patients were discharged with fewer drugs compared to admission than in the usual care cohort (proportion of drugs on admission, relative to discharge (%): usual care vs. intervention: 108.5%, IQR: 100.0–135.8% vs. 92%, IQR: 80.5–103.5%; *p* = 0.002). More RASP PIMs were discontinued in intervention patients during hospital stay, i.e. a mean difference was observed of 1.49 RASP PIMs (95% CI: 0.70, 2.23; *p* < 0.001). Patients in the intervention cohort received furthermore fewer RASP PIMs at discharge (usual care vs. intervention: 2.50, IQR: 2.0–3.8 vs. 1.0, IQR: 0.0–3.0; *p* = 0.008).

None of the individual RASP PIMs had been significantly discontinued more frequently in the intervention than in the usual care cohort. Trends towards higher discontinuation rates in favour of the intervention were observed for the following RASP items: prolonged use of benzodiazepines, zolpidem, zopiclone or zaleplon; prolonged use of antidepressants; use of inhalation corticosteroids in the treatment of COPD GOLD I-II; duplicate therapy (e.g. two different beta blockers). The content of the non-RASP based recommendations was very diverse (i.e. low prevalence per type of recommendation); they could largely be reduced to clinically relevant medication discrepancies and drug therapies lacking or with unclear indications. More information on drug use has been provided in Table 3.

**Table 3:** Outcome measures

	Usual Care (*n* = 29)	Intervention (*n* = 30)	*p*-value
Number of drug-related recommendations (median, IQR)	0.0 (0.0–1.0)	8.0 (6.8–10.0)	< 0.001
Number, based on RASP list (absolute) (median, IQR)	0.0 (0.0–0.0)	3.5 (2.0–5.0)	< 0.001
Proportion of RASP list based recommendation, relative to all provided recommendations (%) (median, IQR)	0.0 (0.0–0.0)	47.2 (33.3–50.0)	< 0.001
Number of other pharmaceutical recommendations, not based on RASP list (median, IQR)	0.0 (0.0–0.0)	3.0 (2.0–5.0)	< 0.001
Number of accepted recommendations by the treating ward-based physician, within 72 h (median, IQR)	0.0 (0.0–0.5)	3.0 (0.0–5.3)	< 0.001
Proportion of accepted relative to all provided recommendations (%) (median, IQR)	0.0 (0.0–25.0)	45.0 (0.0–61.7)	0.010
RASP PIMs at discharge (median, IQR)	2.5 (2.0–3.8)	1 (0.0–3.0)	0.008
Number of drugs at discharge (median, IQR)	8.5 (6.0–11.8)	8.0 (5.0–11.0)	0.404
Proportion of number of drugs at discharge relative to admission (%) (median, IQR)	108.5 (100.0–136.8)	92.0 (80.5–103.5)	0.002
Number of discontinued RASP PIMs during hospital stay (mean, SD)	0.79 (1.34)	2.28 (1.62)	< 0.001

*PIM* potentially inappropriate medications

*RASP* Rationalization of Home Medication by an Adjusted STOPP in Older Patients

The Poisson regression analysis identified two significant determinants for the dependent outcome measure of number of IGCT provided drug-related recommendations: application of the systematic medication review in the intervention cohort coincided with an incidence rate ratio (IRR) of 14.1 (95% CI: 8.3, 23.8, *p* < 0.001) and the number of preadmission drugs with an IRR of 1.06 (95% CI: 1.03, 1.09). No other significant determinants were observed. Model A is the unadjusted model with intervention vs. usual care as the sole determinant. Model B is the adjusted model which shows a small effect of the number of preadmission drugs. Both models have been depicted in Table 4.

**Table 4:** Incidence rate ratios for the number of IGCT-provided drug-related recommendations

Model: variable of interest	*P*-value	IRR	95% CI	AIC
Model A: intervention vs. usual care	< 0.001	15.402	9.141–25.951	207.219
Model B: Intervention vs. usual care^a^	< 0.001	14.068	8.329 – 23.764	195.843

^a^Adjusted for the number of preadmission drugs

*IGCT* interdisciplinary geriatric consultation team

*IRR* incidence rate ratio’s

*CI* confidence interval

*AIC* akaike information criterion

## Discussion

A before-after study was undertaken in a large teaching hospital to increase the number of drug-related recommendations provided by the IGCT. Enrolled patients were older adults who had been admitted to other than acute geriatric wards and in whom a IGCT consultation had been requested. The intervention on top of usual care provided by the IGCT was able to significantly increase the number of drug-related recommendations. In this study, we found a very low number of drug-related recommendations in the before cohort (usual care). This might largely be explained by nurse IGCT members having been the first and predominant contact with the patients. These nurses were not yet accustomed with performing medication reconciliation and review.

The following secondary outcome measures should be considered exploratory. The criteria of the RASP list accounted for 47.2% (IQR: 33.3–55.0%) of the provided recommendations in the intervention cohort, which is in accordance with our previous experiences with the RASP list [2]. A median of 45% of the recommendations was accepted by the ward-based physicians in the intervention cohort, with a significant uptake of the IGCT recommendations into the final discharge letter. The latter was unexpected, given that drug-related recommendations were added to the patient’s file without further oral communication. Ward-based physicians were however found ready to apply the recommendations, which suggests promising results on the applicability of such interventions. We hypothesize that a different approach, with active publicity within the hospital for this type of additional IGCT service, would probably increase the downstream uptake of the drug-related recommendations.

Our results are in line with those found in a previous investigation, in which the intervention had also been based on the use of the RASP list [2]. Both this and the previous study were performed in a geriatric inpatient population and investigated comparable interventions. Several differences should be noted however. First, patients were enrolled from acute geriatric care wards as opposed to non-geriatric care wards. As a consequence, usual care consisted of geriatricians in the previous study, who were already trained in improving drug therapies. Second, patients were on average 2 years older and suffered from worse renal function. Third, the medication review service at the time was done solely by hospital pharmacists and pharmaceutical services were furthermore provided during the complete hospital stay, i.e. from admission to discharge. Nonetheless, both investigations comprised comparable comorbid patients who took nearly identical numbers of preadmission drugs. More importantly, the interventions in both studies led to similar reductions of RASP PIMs at discharge, which further corroborates the applicability of a RASP-based intervention in older inpatients.

Providing a structured medication review, such as the one investigated in this study, can improve the quality of prescribing and might also impact several clinical outcome measures, such as drug-related readmissions [2, 8, 26, 27]. The evidence remains limited however to a small number of positive studies, many of which involved ward-based pharmacists, who were frequently incorporated into multidisciplinary teams [4, 5, 28]. Pharmacist interventions frequently entail providing a medication reconciliation, a medication review, patient education and follow-up after discharge [28]. As team members, pharmacists offer an additional perspective on how to improve drug therapies in older patients and have been identified as suitable candidates to apply medication reviews [5, 28]. A structured medication review, whether or not involving pharmacists, has however not been rigorously been investigated within the context of a CGA [5]. This specific context might offer distinct advantages as opposed to providing an ad hoc medication review. Not only the medical and social, but also functional capabilities are ascertained within the CGA context, supporting individualized drug-related recommendations; frailty, life expectancy, cognitive reserve among others all play a significant role in determining whether certain drug therapies should be initiated, changed or discontinued [29].

To the best of our knowledge, only Dalleur et al. have investigated a comparable IGCT based approach, in which a systematic medication review was also provided as part of the CGA [20]. Enrolled participants had similar profiles, having a comparable age and overall drug use. Their intervention was performed however by geriatricians of the IGCT, who applied a medication review that was based on the STOPP criteria. Hence, both study designs differ regarding the specific screening tool and involvement of the specific health-care professional (trained geriatricians versus clinical pharmacy/medicine students). They concluded that using an explicit screening tool was operational in different settings, including the decentral IGCT setting, and that the intervention resulted in a doubling of PIM reduction, which is comparable to our results.

Following strengths of our study can be noted. Due to the design no teaching contamination could have occurred. With an additional effort, in this case performed by two healthcare professionals-in-training having been provided with limited training themselves, the IGCT care delivery model was able to significantly impact drug prescribing for older inpatients. Time investment of the investigators was limited as well: medication reconciliation and review if applied systematically was not considered to be overly time consuming (i.e. less than 30 min per patient). The external validity is furthermore reassuring as the main intervention was carried by two junior investigators under the supervision of a senior investigator. Also, our trial results are in line with results gathered in previous investigations [2, 20].

Several limitations should however be taken into account. This was an exploratory before-after study (*n* = 59), which means that our results might have been overestimated or be explained by unmeasured confounders. Hence, results should be interpreted with caution. Yet, temporal trends would likely be of no influence, as the study was completed in a two-month period within one hospital. Also, other determinants for the primary outcome measure were found to be of no or at best minimal impact in a predefined regression analysis. Lastly, no heterogeneity was observed across multiple outcome measures (e.g. more recommendations, less drugs, less RASP PIMs). Another major limitation is that no clinically relevant patient outcomes were evaluated. A larger and in particular controlled study with less stringent enrolment criteria should hence be undertaken to confirm our findings in a broader inpatient sample and to investigate the impact of the intervention on clinical outcome measures such as adverse drug reactions or unplanned drug-related hospital readmissions. We also did not follow-up patients after discharge, which limits our conclusions regarding the long-term impact of our intervention. Furthermore, not every hospital has access to trained pharmacists or geriatricians to oversee the training of involved health-care professionals, which might limit the external validity of our findings. The latter could however be mitigated by providing education through e-learning [30, 31].

Finally, the following issues should be targeted in future investigations. First, trained pharmacists can play an important role in an IGCT approach and have only been involved infrequently in this specific setting as can be inferred from the scarcity in the literature. Second, further studies should investigate whether ward-based nurses could undertake a relevant role in the medication review process by screening for potentially inappropriate therapies or aiding and implementing therapy changes for potentially inappropriate therapies or aiding in implementing therapy changes [32]. Previous investigations in different settings have already shown that trained nurses can have a beneficial impact on drug use, e.g. in the outpatient follow-up of heart failure patients or the management of atrial fibrillation patients, both settings with a high degree of polypharmacy [33, 34]. Third, CDSS might play an important role in expanding medication review services in the hospital setting and decreasing therapeutic inertia (e.g. alerting to the inappropriate use of sotalol in heart failure patients, or the use of flecainide in patients with previous coronary ischemia). We eagerly await the results of two ongoing European trials, OPERAM and SENATOR, both incorporating software packages to manage inappropriate drug use in older hospitalized adults [35]. Fourth, more studies should be performed to further confirm the benefits of complementing CGA with structured medication reviews.

## Conclusions

Adding a systematic medication review to a CGA performed by the IGCT may have benefits. A large association was seen in this before-after study between providing a systematic medication review and an increase of drug-related recommendations provided by the IGCT. Regression analysis identified the number of preadmission drugs to be another potential determinant for the number of drug-related recommendations. More RASP PIMs were discontinued during hospital stay in the intervention cohort, which coincided with a relative decrease in the number of drugs. Due to the quasi-experimental design, results should be interpreted with caution.

## Abbreviations

AIC: Akaike information criterion
CGA: Comprehensive geriatric assessment
CI: Confidence interval
CrCL CG: Estimated creatinine clearance by the Cockcroft-Gault equation
eGFR CKD-EPI: Estimated Glomerular Filtration Rate by the Chronic Kidney Disease Epidemiology (Collaboration) equation
IGCT: Interdisciplinary geriatric consultation team
IQR: Interquartile range
IRR: Incidence rate ratio
PIM: Potentially inappropriate medication
RASP: Rationalization of Home Medication by an Adjusted STOPP in Older Patients
SD: Standard deviation
